# Evaluation of cochlear angular orientation in patients with CHARGE syndrome

**DOI:** 10.1007/s00405-026-10195-y

**Published:** 2026-03-20

**Authors:** Ergin Eroğlu, Çağrı Külekçi, Canset Aydın, Levent Sennaroğlu

**Affiliations:** 1 Department of Otolaryngology, Hacettepe University, Ankara, Turkey; 2 Department of Otolaryngology, Atılım University, Ankara, Turkey

**Keywords:** CHARGE syndrome, Cochlea, Rotation, Angle, Cochlear implantation

## Abstract

**Purpose:**

This study aimed to evaluate cochlear angulation in patients with CHARGE syndrome and compare it with individuals with normal cochlear anatomy.

**Methods:**

Eighteen patients with CHARGE syndrome followed for sensorineural hearing loss were retrospectively reviewed and compared with 18 age-matched controls with normal cochlear anatomy. Temporal bone computed tomography (CT) and magnetic resonance imaging were analyzed to assess inner ear malformations and cochlear nerve status. Cochlear angulation was measured on axial CT images by calculating the angle formed between lines passing through the basal turns of both cochleae at the level of the round window. Statistical analyses were performed using chi-square and independent samples t-tests.

**Results:**

Cochlear hypoplasia type III was the most common anomaly in the CHARGE group. Cochlear nerves were frequently hypoplastic or absent, whereas all control ears were normal. Mean cochlear angulation was significantly narrower in the CHARGE group compared with controls (94.49° ± 10.02° vs. 114.02° ± 8.17°, *p* < 0.001).

**Conclusion:**

CHARGE syndrome is associated with significantly altered cochlear angulation, which may contribute to surgical challenges during cochlear implantation. Preoperative recognition of this feature may facilitate safer and more effective surgical planning.

## Introduction

Hall [1] and Hittner [2] et al. reported a congenital syndrome characterized by choanal atresia, colobomatous microphthalmia, cardiac anomalies, hearing impairment, and intellectual disability. The acronym CHARGE—representing *coloboma*,* heart defects*,* atresia of the choanae*,* retardation of growth and/or development*,* genital hypoplasia*,* and ear anomalies and/or deafness*—was first introduced by Pagon et al. in 1981 to describe the characteristic features of the syndrome [3]. The major diagnostic criteria include coloboma, choanal atresia, characteristic ear anomalies, and cranial nerve abnormalities. The minor criteria consist of genital hypoplasia, developmental delay, cardiovascular malformations, orofacial clefts, tracheoesophageal fistula, and a distinctive facial appearance.

In patients with CHARGE syndrome, the estimated prevalence of congenital hearing loss is approximately 1 in 8,500 to 12,000 [4]. In addition to external auditory canal anomalies, inner ear malformations are also frequently observed in these patients [5]. Children with inner ear anomalies typically present with profound hearing loss, often requiring cochlear or brainstem implantation for auditory rehabilitation. During cochlear implantation, identifying and locating the cochlea can be particularly challenging [6]. This difficulty arises because patients with CHARGE syndrome frequently present with semicircular canal anomalies and an abnormal course of the facial nerve [7]. In addition, the position of the cochlea itself should be carefully considered in these patients. We propose that, beyond these factors, the cochlea in CHARGE syndrome may exhibit an abnormal angulation within the petrous bone, which could represent the major obstacle in locating the cochlea during surgery. This is the first study to investigate cochlear positioning in patients with CHARGE syndrome.

The aim of this study is to demonstrate that cochlear angulation in patients with CHARGE syndrome differs from that of individuals with normal anatomy.

## Method

The medical records of 18 patients with CHARGE syndrome who were followed for sensorineural hearing loss at the Department of Otolaryngology, Hacettepe University, were retrospectively reviewed. Prior to the study, approval was obtained from the Non-Interventional Ethics Committee of Hacettepe University Faculty of Medicine (SBA 2025/123).

First, the demographic characteristics of the patients were extracted. In addition, CHARGE-associated comorbidities were identified. The temporal bone computed tomography (CT) and magnetic resonance imaging (MRI) scans were evaluated to analyze inner ear malformations and the status of the cochlear nerve. Subsequently, the patients who underwent cochlear implantation within the last five years were identified during the selection of the control group. Eighteen patients were determined through age and sex matching with the CHARGE syndrome group. Patients with inner ear anomalies, craniofacial anomalies, or a history of head trauma were excluded from the control group. In the control group, 6 of the 18 patients underwent bilateral cochlear implantation. Among these six bilateral cases, three received Med-El^®^ Flex 28, two received bilateral Cochlear^®^ Nucleus 522, and one received bilateral Advanced Bionics^®^ cochlear implants. Among the 12 patients who underwent unilateral implantation, nine received Med-El^®^ Flex 28, two received Cochlear^®^ Nucleus 522, and one received Cochlear^®^ Nucleus 622.

**Fig. 1:**
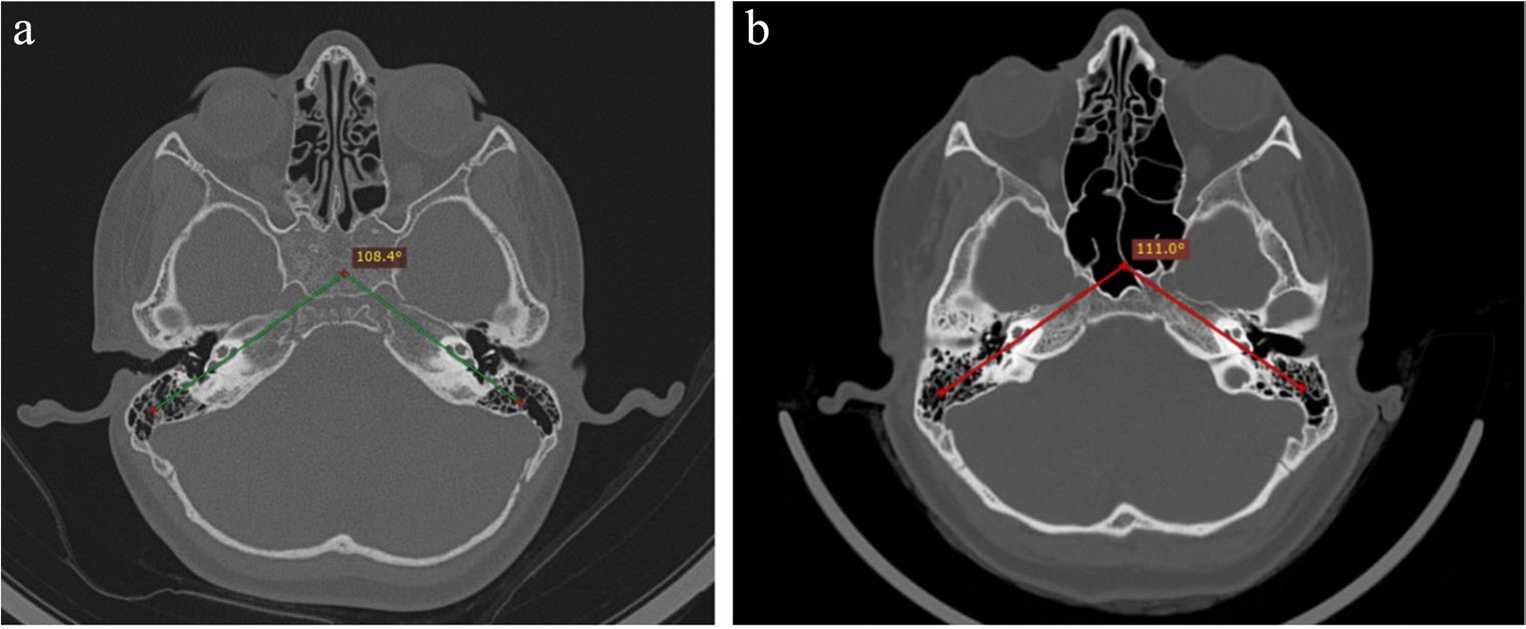
Demonstration of the obtained angles in the two different normal patients (**a**, **b**)

**Fig. 2:**
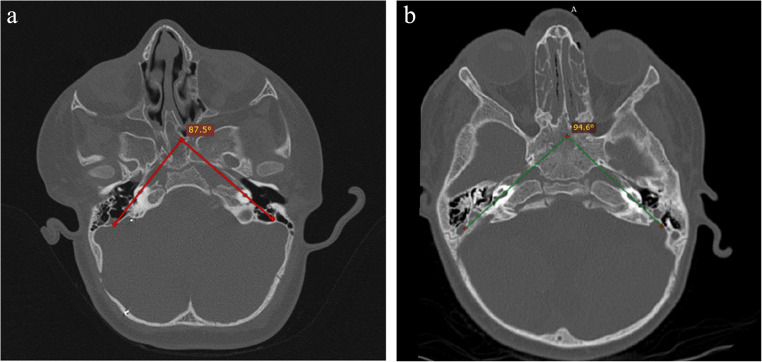
Demonstration of the obtained angles in the patients with Cochlear hypoplasia type-I (**a**) and type-III (**b**)

In the patients’ temporal bone CT scans, both cochleae were aligned within the same plane. The sequence in which the round window niche was clearly visible bilaterally was identified. In this sequence, a line was drawn from the round window niche passing directly over the basal turn of the cochlea. The same procedure was repeated for the contralateral ear. The angle formed by the intersection of these two lines was then recorded (Fig. 1). The identical measurements were performed in the CHARGE group, and the intersection angles were obtained (Fig. 2). The large language model was used solely for English language editing and grammar correction.

### Statistical analysis

Statistical analyses were conducted using IBM SPSS Statistics, version 25.0. Demographic variables were evaluated with frequency distributions and the chi-square test. The distribution of angular values within the groups was assessed using the Shapiro–Wilk test, histogram and other descriptive parameters. Since *p* > 0.05 was obtained for both groups, the data were considered to be normally distributed. The comparisons between groups were performed using the Independent Samples t-test. A p-value of < 0.05 was considered statistically significant.

## Results

In both groups, the male-to-female distribution was observed to be equal. In the CHARGE syndrome patient group, the mean age was determined as 5.58 ± 5.11 years. Among 36 ears, cochlear hypoplasia type III (CH-III) was observed in 24 ears (66.7%), cochlear hypoplasia type I (CH-I) in 5 ears (13.8%), cochlear hypoplasia type II (CH-II) in 3 ears (8.3%), cochlear hypoplasia type IV (CH-IV) in 2 ears (5.5%) and, the cochlear aperture stenosis in 2 ears (5.5%). In 16 patients with magnetic resonance imaging, the cochlear nerve (CN) was hypoplastic in 13 ears (40.6%), absent in 12 ears (37.5%), and normal in 7 ears (19.4%) (Table 1). In the control group, all cochleae and cochlear nerves were observed to be normal.

**Table 1:** The demographic characteristics, inner ear structures, and comorbidities of the patients

Patient number	Age	Gender	Right IEM	Left IEM	Right cochlear nerve	Left Cochlear nerve	Implantation Surgery	Comorbidities
Patient 1	1	M	CH-II	CH-III	Absent	Hypoplasia	Left side Cochlear^®^ Nucleus 522	Coloboma, heart anomaly, retardation, ear anomaly, cranial abnormality
Patient 2	16	F	CH-III	CH-II	Normal	Hypoplasia	N/A	Retardation, genital anomaly, ear anomaly, kidney abNormality
Patient 3	8	M	CH-III	CH-III	N/A	N/A	N/A	Coloboma, retardation, ear anomaly, cranial abnormality
Patient 4	1.5	F	CH-III	CH-III	N/A	N/A	N/A	Heart anomaly, choanal atresia, retardation, ear anomaly
Patient 5	3	M	CH-I	CH-II	Absent	Hypoplasia	N/A	Retardation, ear anomaly, cleft palate, hypothyroidism, cranial abnormality
Patient 6	1.5	M	CH-I	CH-III	Absent	Hypoplasia	Right side Cochlear^®^ 24 M ABI + left side Cochlear^®^ Countour Advance	Heart anomaly, retardation, ear anomaly, esophageal atresia
Patient 7	11	M	CH-I	CH-III	Absent	Hypoplasia	N/A	Heart anomaly, retardation, genital anomaly, ear anomaly,
Patient 8	5	F	Cochlear aperture stenosis	Cochlear aperture stenosis	Normal	Normal	N/A	Coloboma, heart anomaly, retardation, ear anomaly
Patient 9	12	M	CH-III	CH-III	Normal	Hypoplasia	Right side Cochlear^®^ Nucleus 422	Heart anomaly, retardation, genital anomaly, ear anomaly, cleft palate
Patient 10	16	F	CH-III	CH-III	Normal	Absent	N/A	Retardation, genital anomaly, ear anomaly, esophageal atresia
Patient 11	3	M	CH-IV	CH-IV	Normal	Hypoplasia	Right side Cochlear^®^ Nucleus 522	Coloboma, heart anomaly, retardation, ear anomaly, cleft palate
Patient 12	9	F	CH-III	CH-III	Hypoplasia	Absent	Right side Med-El^®^ Form 19	Heart anomaly, retardation, ear anomaly, esophageal atresia
Patient 13	2	F	CH-I	CH-I	Absent	Absent	Bilateral Med-El^®^ Concerto ABI	Coloboma, heart anomaly, retardation, ear anomaly, kidney abNormality
Patient 14	4	F	CH-III	CH-III	Absent	Absent	Right side Med-El^®^ Concerto ABI	Coloboma, heart anomaly, retardation, ear anomaly
Patient 15	2	F	CH-III	CH-III	Absent	Absent	Left side Med-El^®^ Concerto ABI	Coloboma, heart anomaly, retardation, ear anomaly, hypothyroidism
Patient 16	3	F	CH-III	CH-III	Hypoplasia	Hypoplasia	Right side Med-El^®^ Form 19	Heart anomaly, retardation, ear anomaly, cleft palate, urinary system anomaly
Patient 17	1.5	M	CH-III	CH-III	Hypoplasia	Hypoplasia	N/A	Retardation, genital anoamaly, ear anomaly, cleft palate, urinary system anomaly
Patient 18	1	M	CH-III	CH-III	Normal	Hypoplasia	Right side Med-El^®^ Form 19	Heart anomaly, retardation, ,genital anomaly, ear anomaly, cleft palate

*M* male, *F* female, *IEM* Inner ear malformation, *CH* Cochlear Hypoplasia, *CI* Cochlear implantation, *ABI* Auditory brainstem implantation

In the group followed for CHARGE syndrome, six patients underwent unilateral cochlear implantation, one patient underwent bilateral auditory brainstem implantation, two patients underwent unilateral auditory brainstem implantation, and one patient underwent simultaneous cochlear and auditory brainstem implantation. The posterior timpanotomy approach was used for middle ear access in cochlear implantation.

In the CHARGE syndrome patient group, computed tomography measurements revealed a mean angle of 94.49° ± 10.02°. The minimum angle was 76.7°, the maximum was 114.1°, and the median was 94.2°. In the control group, the mean angle was found to be 114.02° ± 8.17°, with a minimum of 98.4°, a maximum of 132°, and a median of 112.75°. A statistically significant difference was observed between the angle values of the two groups (*p* < 0.001) (Table 2).

**Table 2:** Comparison of Cochlear Angles Values Among Groups

Anomaly type	Axial Angles		
	Mean ± SD	Median (Min-Max)	p value (95% CI)
CHARGE Syndrome Group	94,48°±10,02°	94,2° (76,7-114,1)	< 0.001 (4,59 − 13,79)
Control Group	114,02°±8,17°	94,2° (98,4-132)	

*SD* Standart deviation, *CI* Confidence interval

## Discussion

Inner ear anomalies accompanying CHARGE syndrome have previously been described [8]. The technical challenges of cochlear implantation surgery in these patients have also been well recognized [9]. However, the underlying reasons for these surgical difficulties have not been fully elucidated. In this patient population, detailed evaluation of the cochlear position within the temporal bone revealed that the angular rotation of the cochlea was abnormal. To the best of our knowledge, this is the first study in the literature to demonstrate abnormal cochlear orientation in patients with CHARGE syndrome.

Ear anomalies are frequently observed in patients with CHARGE syndrome. Although the embryologic development patterns of the auricle, external, middle, and inner ear differ, anomalies can be found in all four structures in CHARGE syndrome. The auricle may present as short and broad, with small or absent lobes, or with one missing crus [10]. In the middle ear, defects may be seen in the manubrium of the malleus or the long process of the incus [11]. In the inner ear, various abnormalities ranging from semicircular canal agenesis to cochlear malformations have been reported [12].

Sennaroglu and colleagues classified inner ear anomalies into eight different groups [13]. Inner ear anomalies are frequently associated with CHARGE syndrome. In the study by Szleper et al. [14], CH-III was the most common type, observed in 60% of 10 patients, while in the study by Monsanto et al., it was seen in 75% of 6 patients [15]. In patients with CHARGE syndrome, the bony cochlear nerve canal (BCNC) may appear normal, hypoplastic, or aplastic. In our study, bilateral aplasia of the bony cochlear nerve canal was observed in one patient. In the study conducted by Monsanto et al., a narrow BCNC was reported in 7 ears [15]. In the study by Holcomb et al. involving 15 patients, the BCNC was found to be normal in 47%, hypoplastic in 23%, and absent in 30% of cases [16]. In addition, histopathological studies have reported cases in which spiral ganglion cells were completely absent [17]. Another important aspect when evaluating inner ear structures is the presence of the cochlear nerve. Holcomb et al. did not observe the cochlear nerve in 8 of 16 ears (50%) [16], while Monsanto et al. reported its absence in 2 of 12 ears (16.6%) [15]. Radiological imaging plays a crucial role in the evaluation of hearing loss in patients with CHARGE syndrome. Inner ear anomalies, the modiolus, and BCNC should be assessed using temporal bone computed tomography, while the presence of the cochlear nerve should be determined through magnetic resonance imaging. Following these steps, the decision between cochlear implantation and auditory brainstem implantation must be made with great care in the management of these patients.

In patients with CHARGE syndrome, hearing loss of varying degrees is observed in 60–100% of cases [18, 19]. Approximately 70% of patients with CHARGE syndrome require hearing rehabilitation by the age of five [20]. The type and severity of hearing loss in these patients can range from conductive hearing loss due to external auditory canal anomalies to profound sensorineural hearing loss associated with the absence of the cochlear nerve [21]. Patients with bilateral profound sensorineural hearing loss who meet the criteria are considered candidates for cochlear implantation. Each step of cochlear implantation surgery may present unique challenges. In the study by Birman et al., all ten CHARGE patients had small mastoids, and the lateral semicircular canal was absent [6]. In four patients, the cochlea was very difficult to locate, while in three others, the initial surgery had to be aborted because the cochlea could not be found. The round window was reported to be positioned posteriorly. Inner ear malformations alone do not fully explain the difficulty in locating the cochlea in these patients. We also identified differences in the spatial orientation of the cochlea compared to normal individuals. In our study, we found that the cochlea was positioned at a different angular orientation relative to the axis of the petrous bone. This abnormal orientation may contribute to the difficulty in identifying the round window and complicate the cochlear implantation procedure.

The cochlea occupies a complex spatial position within the petrous bone. The main reason for this difficulty is the reliance on two-dimensional interpretation of radiologic images. The basal turn and the round window are the most important landmarks in cochlear implantation surgery. Previous studies have used the basal turn or the round window membrane as reference points for assessing cochlear orientation [22, 23]. In our study, the mean sum of the angles formed by both cochleae with the midsagittal plane in the normal population was found to be 114.02°. In the study conducted by Tang et al., this angle—defined as the beta angle—was measured as an average of 55.74 ± 3.50° for the left ear and 57.84 ± 0.45° for the right ear [24]. In the CHARGE syndrome patient group, a narrower angle was observed. This finding is attributed to an increased lateral rotation of the anterior part of the cochlea within the petrous bone on the axial plane compared to normal anatomy. The labyrinthine morphology has traditionally been considered stable after birth, Lloyd et al. observed a statistically significant decrease in the angulation (“rotation”) of the basal turn relative to the midsagittal plane with increasing age [25]. To eliminate this potential confounding factor, the CHARGE syndrome group and the control group were matched for age.

In this study, the primary limitation was that the measurements were performed two-dimensionally using computed tomography. Considering the cochlea’s coiled and complex three-dimensional structure, it is possible that, in addition to rotational movement, other angular variations may also occur. Defining such variations with the currently available imaging techniques is difficult. Furthermore, in patients with CHARGE syndrome, the presence of cochlear hypoplasia makes accurate positioning and measurement of the cochlea considerably more challenging compared to the control group. Finally, the small number of patients represents another limitation of the study.

## Conclusion

This study demonstrated that patients with CHARGE syndrome exhibit a significantly narrower cochlear angulation compared to individuals with normal anatomy. The altered spatial orientation of the cochlea within the petrous bone may contribute to the surgical challenges encountered during cochlear implantation in this population. Recognizing these anatomical differences preoperatively can assist surgeons in planning safer and more effective implantation strategies.
